# Distinct Cargos of Small Extracellular Vesicles Derived from Hypoxic Cells and Their Effect on Cancer Cells

**DOI:** 10.3390/ijms21145071

**Published:** 2020-07-17

**Authors:** Geoffroy Walbrecq, Christiane Margue, Iris Behrmann, Stephanie Kreis

**Affiliations:** Department of Life Sciences and Medicine (DLSM), University of Luxembourg, 6, avenue du Swing, L-4367 Belvaux, Luxembourg; Geoffroy.walbrecq@uni.lu (G.W.); christiane.margue@uni.lu (C.M.); iris.behrmann@uni.lu (I.B.)

**Keywords:** exosomes, extracellular vesicles, cancer, hypoxia, TME, immunity, biomarker

## Abstract

Hypoxia is a common hallmark of solid tumors and is associated with aggressiveness, metastasis and poor outcome. Cancer cells under hypoxia undergo changes in metabolism and there is an intense crosstalk between cancer cells and cells from the tumor microenvironment. This crosstalk is facilitated by small extracellular vesicles (sEVs; diameter between 30 and 200 nm), including exosomes and microvesicles, which carry a cargo of proteins, mRNA, ncRNA and other biological molecules. Hypoxia is known to increase secretion of sEVs and has an impact on the composition of the cargo. This sEV-mediated crosstalk ultimately leads to various biological effects in the proximal tumor microenvironment but also at distant, future metastatic sites. In this review, we discuss the changes induced by hypoxia on sEV secretion and their cargo as well as their effects on the behavior and metabolism of cancer cells, the tumor microenvironment and metastatic events.

## 1. Introduction

**Figure 1:**
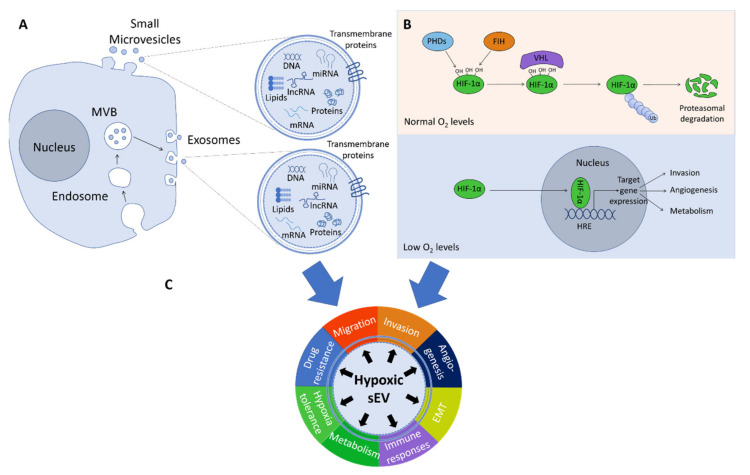
Overview of small extracellular vesicles (sEVs), their content, mechanisms of hypoxia and their potential biological roles. (**A**) sEVs are constituted of exosomes and small microvesicles. Exosomes are secreted after fusion of multivesicular bodies (MVB) with the plasma membrane and microvesicles are released by direct budding from the plasma membrane. sEVs can carry DNA fragments, mRNAs, microRNAs, lncRNAs, proteins, lipids and all other biological molecules. (**B**) Under normal O_2_ availability, prolyl hydroxylase domain proteins (PHDs) hydroxylate proline residues on HIF-1α, which triggers the binding of the von Hippel-Lindau (VHL) tumor suppressor protein to HIF-1α leading to ubiquitination and degradation of HIF-1α. Under hypoxia, HIF-1α is stabilized and binds hypoxic response elements (HRE), thereby triggering target gene expression. This leads to changes in the metabolism of the cells and may stimulate invasion and angiogenesis. (**C**) Hypoxic sEVs play roles in invasion, migration, angiogenesis, epithelial to mesenchymal transition (EMT) and drug resistance of cancer cells. They also regulate immune responses, metabolism and hypoxia tolerance of target cells, details of which are discussed below.

Extracellular vesicles are defined as particles, which are delimited by a lipid bilayer and which cannot replicate [1]. They can be subdivided according to their size into small EVs (≤200 nm; sEVs) and medium/large EVs (> 200 nm; lEVs) [1]. Both sEV and lEVs have various roles in normal physiology, notably in development, differentiation, angiogenesis, coagulation, immune modulation, organ homeostasis and maternal-fetal communication [2]. EVs encompass microvesicles and larger vesicles such as apoptotic bodies (500 ≤ 2000 nm), released as bulges from apoptotic cells and large oncosomes (1 ≤ 10 µm), which are derived by the membrane shedding of cancer cells [1,3]. Large oncosomes have various roles in cancer growth and progression and apoptotic bodies may be involved in intercellular communication and immune modulation [4,5]. On the other hand, the group of sEVs also covers exosomes (≤200 nm) derived from late endosomes and small microvesicles (≤200 nm), which are plasma membrane-derived particles [1] (Figure 1). Microvesicles biogenesis occurs by outward budding of the plasma membrane and exosome biogenesis takes place by inward budding of multivesicular bodies (MVB) with the plasma membrane and this fusion results in the formation of intraluminal vesicles (ILV), which are then released into the extracellular medium [6]. Both exosomes and microvesicles contain DNA, RNA, proteins, lipids and metabolites. RNA molecules found in these structures include microRNAs, mRNAs, long non-coding RNAs (lncRNAs) and circular RNAs [6]. Metabolites found in sEVs consist of amino acids, nucleotides, nucleosides, organic acids, sugars, alcohols, vitamins and derivatives from lipids (reviewed in [7]). Several databases have been established recently, which cover the protein and RNA content of exosomes and microvesicles, such as Exocarta, Vesiclepedia and EVpedia [8,9,10]. Since methods are missing to clearly separate exosomes from small microvesicles (≤200 nm) due to their identical densities [1], this review will focus on both types, named hereafter small extracellular vesicles (sEVs).

sEVs can deliver their messages into the target cell by several distinct mechanisms: through non-specific processes such as endocytosis (by macropinocytosis or micropinocytosis [6,11]) or through the interaction of membrane proteins expressed at the sEV surface with receptors at the surface of the target cell (reviewed in [6]). The receptor-dependent pathway can involve either a protein, a lipid or a sugar at the sEV surface [6]. Once the sEV has entered the cell, the current theory is that the vesicle is fusing with the endosome to release its cargo, which can then be delivered to other compartments of the cell such as the endoplasmic reticulum or the nucleus [6,12,13].

Tumor sEVs were shown to educate cells at pre-metastatic sites towards a pro-metastatic phenotype. Thus, sEVs can help cancer cells to metastasize to a new organ [14]. Although there is no evidence for an indispensable receptor at the surface of the acceptor cell, Hoshino et al. demonstrated that integrins expressed at the surface of the sEVs could determine in which organ the metastasis would occur [15]. It is known that hypoxia can upregulate the expression of some integrins. However, it remains to be shown whether hypoxia-induced changes of integrin expression at the surface of sEVs might influence the location of tumor metastasis [16,17].

Hypoxia is a common feature in many solid cancers and is defined by a lower oxygen tension compared to physiological conditions. The oxygen tension found in normal tissues is about 30–60 mm Hg, but can reach low values of around 10 mm Hg in skeletal muscle or in skin [18], while the median value in tumor cores is ranging between 2 and 16 mm Hg [19]. Intra-tumoral hypoxia is caused by the lack of blood vessels and the fast growing rate of cancer cells, which adapt to this low tissue oxygenation by activating the hypoxia-inducible transcription factors (HIFs). The two main HIFs upregulated under hypoxia are HIF-1α and HIF-2α. Under normoxic conditions, prolyl hydroxylase domain proteins (PHDs) hydroxylate proline residues on HIF-1α, which allows the binding of von Hippel-Lindau (VHL) tumor suppressor protein to HIF-1α for subsequent ubiquitination. The ubiquitinated HIF-1α is then degraded by the 26 S proteasome [19]. In addition, factor inhibiting HIF1 (FIH-1) hydroxylates HIF-1α on an asparagine residue, which inhibits the interaction of HIF-1α with its coactivators [19]. Under low oxygen availability, the activity of both PHD and FIH-1 is inhibited, leading to the stabilization and translocation of HIF-1α to the nucleus. HIF-1α can subsequently trigger the transcription of multiple target genes, e.g., those encoding vascular endothelial growth factor, carbonic anhydrase 9 or glucose transporter 1 [19]. HIF-1α is stabilized during acute hypoxia, while under prolonged hypoxia, HIF-2α is activated [20]. The transcriptional responses mediated by HIFs then activate processes involved in angiogenesis, invasion and in the metabolic adaptation of cancer cells.

Hypoxia influences the uptake of sEVs. Jung et al. recently demonstrated that hypoxic breast cancer cells preferentially take up hypoxic sEVs compared to normoxic sEVs [21]. This was confirmed using sEVs loaded with anticancer drugs to treat hypoxic cancer cells; hypoxic sEVs were more efficient to kill hypoxic cancer cells compared to normoxic sEVs [21]. In a previous study, we did not see significant differences in the uptake of normoxic sEVs between melanoma cells grown under normoxia or hypoxia, but we observed that cells produced more exosomes under hypoxic conditions [22]. Hypoxia generally induces an increase of sEV secretion from cancer cells [22,23,24] as well as a change in their cargo, which reflects the status of the cells from which they are derived [22,25,26]. Rab22 a, a small GTPase and a target gene of HIF-1α and HIF2α, is involved in the production of sEVs [27]. In addition, various stresses encountered by cells under hypoxia, like oxidative stress or low pH, can also increase sEV secretion [28,29,30,31]. Very recently, Patton et al. showed that hypoxia changed the size distribution of sEVs with a clear trend toward smaller average vesicle size. In pancreatic cancer cells, hypoxia promoted a time-dependent release of sEVs and only a minimal induction of medium EVs (mEVs) and large EVs (lEVs) [32].

The growth and progression of cancer cells is crucially affected by their interaction with the surrounding cells in their niche [33]. This niche, or tumor microenvironment (TME), is where cancer cells interact with stromal cells (fibroblasts and endothelial cells) and immune cells, including natural killer (NK) cells, dendritic cells, myeloid-derived suppressor cells, T cells and macrophages [33]. Cancer cells and cells from the TME communicate through direct contact between cells, by releasing cytokines and other soluble factors as well as sEVs [33].

In this review, we will cover the role of hypoxic sEVs produced by either cancer cells or cells from the TME. We will focus on the role of miRNAs and proteins that have been identified in hypoxic EVs and their possible effects on migration, invasion, angiogenesis, epithelial to mesenchymal transition (EMT), immune response pathways, metabolism and resistance to drug treatment.

## 2. Hypoxic sEVs’ Cargo and Its Role in Key Biological Processes Related to Cancer

### 2.1. Migration and Invasion of Cancer Cells

The protein cargo of hypoxic cancer sEVs can influence migration, invasion and metastasis of cancer cells. Matrix metalloproteinase (MMP), especially MMP2, MMP9 and MMP14 activity is associated with invasion and metastasis of tumor cells (reviewed in [34]). Metalloproteinases degrade the extracellular matrix (ECM) and thus enhance the invasion of cancer cells [34]. Interestingly, MMP2, MMP9 and MMP13 levels were increased in sEVs derived from hypoxic prostate cancer cells and hypoxic nasopharyngeal carcinoma cells [35,36] and exosomal MMP13 has been shown to enhance migration and invasion of recipient cells in vitro and in vivo [36]. MMP2 and MMP9 activity and a number of signaling molecules like transforming growth factor β (TGF-β), tumor necrosis factor α (TNF-α) and interleukin 6 (IL-6) were increased in hypoxic (compared to normoxic) sEVs [35]. In endothelial cells, MMP2 activity has been shown to be induced by hypoxic sEVs derived from renal carcinoma cells [37] while MMP14 expression levels were found to be increased in sEVs from hypoxic pancreatic cells together with C4.4A and α6β4 integrin [38]. C4.4A, a structural homologue of the urokinase receptor, is enriched in hypoxic sEVs in a HIF-1α-independent way and the association of C4.4A with α6β4 integrin and MMP14 leads to an increase of motility, due to laminin degradation [38]. In addition, lysyl oxidase (LOX) enzymes, also found enriched in hypoxic sEVs [39], catalyze an important step of the crosslinking of collagen and also elastin, increasing the stiffness of the ECM and thereby facilitating cancer cell adhesion and invasion into the ECM (reviewed in [40]). Furthermore, in ovarian cancer, hypoxic sEVs enriched in CD171 promote cell migration and trigger extracellular signal-regulated kinase (Erk) phosphorylation [41]. Hypoxic sEVs derived from colorectal cancer cells contain Wnt4 and promote metastasis of normoxic colorectal cancer cells by enhancing β-catenin translocation to the nucleus and subsequent activation of the β-catenin signaling pathway, increasing the migratory and invasive properties of colorectal cancer cells [42]. Taken together, metalloproteinases and other enzymes modulating the TME, which are transported by sEVs, clearly have an impact on migration and invasion of cancer cells, thereby modulating their metastatic potential.

In addition to the proteins mentioned above, miRNAs loaded in hypoxic sEVs are also involved in the transfer of pro-metastatic properties. sEVs derived from hypoxic hepatocellular carcinoma cells deliver miR-1273f, which increases proliferation and metastasis by targeting LIM Homeobox 6 (LHX6), an inhibitor of the Wnt/β-catenin pathway [43]. miR-21, secreted in hypoxic sEVs from oral squamous carcinoma cells, promotes migration and invasion of normoxic cells [24].

**Figure 2:**
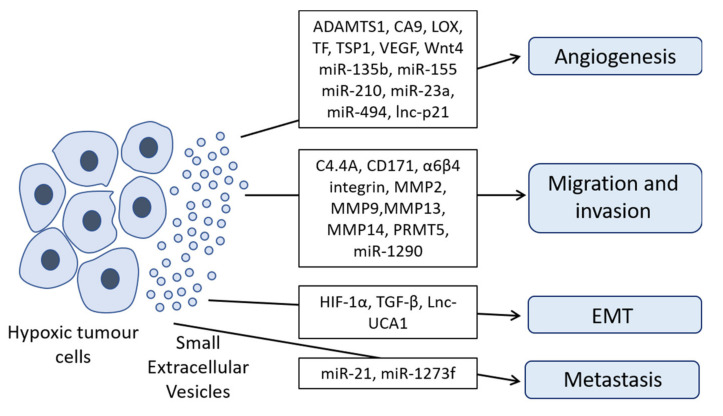
Hypoxic sEVs promote progression and metastasis of cancer cells. Hypoxic sEVs enriched with a disintegrin and metalloproteinase with thrombospondin motifs 1 (ADAMTS1), carbonic anhydrase 9 (CA9), protein-lysine 6-oxidase (LOX), tissue factor (TF), thrombospondin-1 (TSP1), vascular endothelial growth factor (VEGF), Wnt4, miR-135b, miR-155, miR-210, miR-23a, miR-494 and/or lnc-p21 promote angiogenesis. Hypoxic sEVs loaded with C4.4A, CD171, α6β4 integrin, matrix metalloproteinases (MMP2, MMP9, MMP13 and MMP14), protein arginine methyltransferase 5 (PRMT5) and/or miR-1290 drive migration and invasion of target cancer cells. HIF-1α, TGF-β and/or Lnc-UCA1 contained in hypoxic sEVs can enhance epithelial to mesenchymal transition (EMT). miR-21 and/or miR-1273f, secreted in hypoxic sEV, promote metastasis of cancer cells.

An overview of the effects of hypoxic sEVs on key biological processes in the recipient cells through transfer of oncoproteins, miRNAs and lncRNAs is presented in Figure 2. A summary of the role of proteins contained in hypoxic sEVs on migration, invasion, angiogenesis and immune response pathways is provided in Table 1.

### 2.2. Angiogenesis

HIF-1α and HIF-2α knockout mice show lethal defects in vascularization, demonstrating an important role for HIFs in angiogenesis [51,52,53,54]. Hypoxic sEVs can promote angiogenesis through delivery of their cargo to various cells of the TME, affecting the phenotype and the transcriptome of endothelial cells [55]. Various proteins carried by hypoxic sEVs are involved in the promotion of angiogenesis, such as protein-lysine 6-oxidase (LOX), thrombospondin-1 (TSP1), vascular endothelial growth factor (VEGF) and a disintegrin and metalloproteinase with thrombospondin motifs 1 (ADAMTS1), Wnt4, tissue factor (TF) and CA9 (carbonic anhydrase 9) [37,39,42,44]. LOX levels, together with TSP1, VEGF and ADAMTS1 were found elevated in sEVs derived from hypoxic glioblastoma cells, driving angiogenesis-related processes in endothelial progenitor cells [39]. Hypoxic colorectal cancer cells transfer Wnt4 via sEVs to endothelial cells where it activates the β-catenin signaling pathway, also promoting angiogenesis [42]. TF, which is secreted into sEVs derived from hypoxic glioma cells, triggers angiogenesis in human umbilical vein endothelial cells by activating protease activate receptor 2 (PAR2), triggering subsequent Erk phosphorylation [44]. Finally, CA9, whose levels were enriched in sEVs derived from hypoxic renal cell carcinoma, stimulates migration and tube formation of human umbilical vein endothelial cells [37].

Several miRNAs secreted in sEVs derived from hypoxic cancer cells have been shown to be involved in angiogenesis. miR-135b contained in sEVs derived from hypoxic drug-resistant myeloma cells, targets FIH-1 in endothelial cells, thus increasing the activity of HIF-1α and promoting angiogenesis [56]. In lung cancer, miR-23a and miR-494 within hypoxic sEVs were also shown to promote angiogenesis [57,58]. mir-23a inhibits expression of the prolyl hydroxylase and tight junction protein ZO-1, thereby enhancing angiogenesis [57]. mir-494 targets the phosphatase and tensin homolog protein (PTEN), a negative regulator of the PI3K/Akt pathway, thus increasing levels of phosphorylated Akt (p-Akt). P-Akt subsequently phosphorylates the endothelial nitric oxide synthase (eNOS), leading to an increase of angiogenesis in endothelial cells [58]. sEVs from hypoxic leukemia cells are enriched with miR-210, the “prototype” of hypoxia-associated miRNAs, inhibiting Ephrin-A3 expression in endothelial cells and leading to an increase in tube formation [59]. Likewise, miR-155, enriched in sEVs of hypoxic hepatocellular carcinoma cells, also induces angiogenesis [60]. Apart from the well-known miR-210, we recently identified miR-1290 as a novel hypoxia-associated miRNA, which was highly abundant in hypoxic melanoma sEVs. On the other hand, miR-23a-5p and -23b-5p were consistently downregulated in hypoxic conditions, while the protein levels of the miR-23a/b-5p-predicted target *IPO11* were concomitantly upregulated [22]. Furthermore, lncRNAs can be transferred: hypoxic non-small lung cancer cells transfer lncRNA-p21 via sEVs to endothelial cells, promoting tube formation and tumor cell adhesion [61]. Overall, a number of miRNAs and lncRNAs contained in hypoxic sEVs actively shape the migration, invasion, angiogenesis and immune response pathways (summarized in Table 2). Of note, only a small proportion of the total cellular miRNA pool is encapsulated into sEVs. In this context, Chevillet et al. analyzed the number of miRNA molecules per sEV isolated from plasma and found that there is less than one molecule of a particular miRNA per sEV [62]. One additional study confirmed a low ratio of miRNAs per sEV while another stoichiometric analysis found more than 10 copies of a given miRNA per sEV [63,64]. However, the high amount of sEVs secreted by cells in pathophysiological states and/or under hypoxia may compensate for the potentially low number of miRNAs molecules, still providing functionally relevant amounts of a given miRNA or family of miRNAs to the recipient cells.

### 2.3. Epithelial Mesenchymal Transition

Epithelial to mesenchymal transition (EMT) is an important process by which cancer cells evade their original niche and subsequently invade and migrate towards other tissues [77]. During EMT, cells lose their epithelial characteristics and gain mesenchymal features [77]. Those changes are accompanied by the loss or down-regulation of E-cadherin and the increase of β-catenin expression [77]. Loss of E-cadherin leads to the disruption of cell-to-cell contacts, and other cytoskeletal alterations [77]. In this context, Ramteke et al. reported that hypoxic sEVs repress the expression of E-cadherin in normoxic target cells, thus promoting EMT [35]. HIF-1α secreted in sEVs from nasopharyngeal carcinoma cells was also found to trigger EMT-related processes in recipient cells [46] and signaling molecules such as TGF-β, transported by hypoxic sEVs [35], support EMT through induction of chromatin changes (reviewed in [78]). Finally, the lncRNA UCA1, in sEVs of hypoxic bladder cancer cells, promotes EMT in vitro and in vivo [74].

sEVs do not only carry proteins and RNAs, but also lipids [79]. Hypoxia has been shown to induce lipid accumulation in cells and sEVs released by hypoxic cancer cells supporting growth and invasiveness of hypoxic prostate cancer cells following re-oxygenation [80]. Given the large variety of lipid species, further studies will be needed to investigate the role of lipids contained in sEVs derived from hypoxic cancer cells or cells from the TME on the progression of cancer in more detail.

### 2.4. Immune Response Pathways

Hypoxia has been known to play a role in the progression of cancer cells by suppressing the response of the immune system and by altering the differentiation of immune cells [81], and hypoxic sEVs are involved in mediating those effects. For example, miR-10 and miR-21 secreted by hypoxic sEVs derived from glioma cells target RAR-related Orphan Receptor α (Rorα) and PTEN expression, respectively, in order to repress the myeloid-derived suppressor cells [65].

Macrophages can differentiate into two main subpopulations: the pro-inflammatory M1 macrophages and the anti-inflammatory M2 macrophages, which promote tumor growth [82]. In a non-cancerous microenvironment, hypoxia can promote M1 polarization. This is in contrast to the glioma microenvironment, where hypoxia has been shown to support M2 polarization by upregulating TGF-β and macrophage colony-stimulating factor receptor (MCSFR) [83,84]. Although there is still no consensus whether hypoxia promotes M1 or M2 polarization, there are now several reports showing that hypoxic sEVs promote M2 polarization. miR-1246, enriched in sEVs derived from hypoxic glioma cells, targets the telomeric repeat-binding factor 2-interacting protein 1 (TERF2IP), which subsequently downregulates the NFκB pathway and activates the STAT3 pathway, leading to M2 macrophage polarization [66]. miR-301a-3p levels were increased in hypoxic pancreatic cell-derived sEVs and transfer of miR-301a-3p to macrophages elicits an M2 phenotype through activation of the PTEN/PI3Kγ signaling [68]. sEVs of hypoxic melanoma, squamous skin carcinoma and lung cancer cells are loaded with immunosuppressive proteins like colony-stimulating factor 1 (CSF-1), C-C motif chemokine 2 (CCL2), ferritin heavy chain (FTH), ferritin light chain (FTL) and TGF-β as well as the miRNA let-7a [47]. The delivery of let-7a increases oxidative phosphorylation and M2 polarization of targeted macrophages through the downregulation of the insulin-Akt-mTOR pathway by inhibiting expression of the insulin receptor substrate 1 (IRS1), insulin receptor substrate 2 (IRS2), insulin receptor (INSR) and insulin-like growth factor 1 receptor (IGF1R), being involved in the insulin signaling pathway [47]. Furthermore, miR-21-3p, miR-125b-5p, miR-181d-5p and miR-940 are loaded in sEVs derived from hypoxic ovarian cancer cells and also induce the polarization of macrophages towards a tumor-like M2 phenotype [67,69] by targeting the SOCS4/5/STAT3 signaling pathway [69]. The lncRNA BRCT1, enriched in breast cancer sEVs was also shown to promote an M2 phenotype in macrophages [75] and finally, sEVs derived from hypoxic mesenchymal stem cells transfer miR-21-5p, which inhibits PTEN expression levels, subsequently promoting the differentiation of macrophages towards an M2 phenotype [85].

Natural killer (NK) cells are innate lymphoid cells, which have a strong anti-tumor activity [81]. NK cell function is impaired under hypoxia, which induces a decrease of the expression of several NK cell receptors responsible for the killing of target cells [81,86]. Berchem et al. have shown that the transfer of hypoxic tumor sEVs carrying TGF-β and miR-23a has immunosuppressive effects on NK-cells [48]. TGF-β downregulates the expression of NKG2D, an activating receptor of NK cells while miR-23a targets CD107a, a marker of NK cell degranulation [48]. Maus et al. have described that hypoxic melanoma sEVs regulate dendritic cell maturation and affect the cytokines and chemokines released by dendritic cells [87]. Those studies demonstrate that hypoxia induced-sEVs can alter NK cell function.

γδ T cells, a particular class of T cells containing T cell receptors (TCRs) and TCR δ chains, also develop a cytotoxic activity towards cancer cells by recognizing antigens without requiring their presentation on major histocompatibility complexes. However, in certain cases, they may have pro-tumoral activities by secreting inflammatory factors such as IL-4, IL-10 and the C-X-C motif chemokine 13 (CXCL13) [88,89]. In this context, sEVs derived from hypoxic oral cancer cells were shown to inhibit the expansion and cytotoxicity of γδ T cells, while normoxic sEVs promote it [70]. Interestingly, secretion of perforin, granzyme B and IFN-γ by γδ T cells was decreased after treatment with hypoxic cancer sEVs via a mechanism involving exosomal miR-21 [70]. The authors demonstrated that miR-21 exerts its action in myeloid-derived suppressor cells that have a suppressive role towards γδ T cells, by inhibiting PTEN signaling and decreasing the expression of the programmed death-ligand 1 (PD-L1) at their surface [70]. Furthermore, transfer of sEVs derived from hypoxic nasopharyngeal carcinoma cells impairs T cell proliferation and differentiation of Th1 and Th17 cells, while it enhances the differentiation of regulatory T cells (T-reg). This impairment is due to the decrease of fibroblast growth factor 11 (FGF11) expression levels, which is targeted by the exosomal miR-24-3p [71]. Rong et al. recently showed that sEVs secreted by hypoxic breast cancer cells inhibit T cell proliferation via TGF-β expressed at the surface of hypoxic sEVs [49].

**Figure 3:**
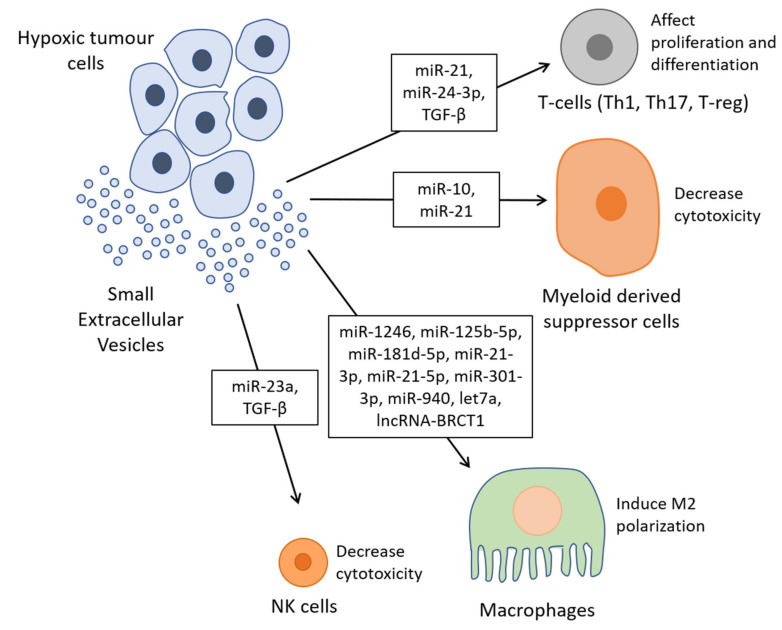
Hypoxic sEVs modulate the immune system. miR-23a and TGF-β, loaded in hypoxic sEVs, decrease cytotoxicity of natural killer (NK) cells. The transfer of miR-1246, miR-125b-5p, miR-181d-5p, miR-21-3p, miR-21-5p, miR-301-3p, miR-940, let7a and/or lncRNA BRCT1 by hypoxic sEVs induce M2 macrophage polarization. Hypoxic sEVs loaded with miR-10 and miR-21 decrease cytotoxicity of myeloid derived suppressor cells. Hypoxic sEVs affect the proliferation of T cells and differentiation of Th1, Th17 and regulatory T cells (T-reg) by transferring miR-21, miR-24-3p and TGF-β.

Altogether, these findings indicate that sEVs derived from hypoxic cells are implicated in the downregulation of immune cells, which ultimately benefits cancer cells (Figure 3, Table 1 and Table 2). 

### 2.5. Metabolism and Hypoxic Tolerance

Under hypoxia, cells undergo changes in their metabolism, which are elicited by HIF1-α. In activated hepatic stellate cells, HIF1-α can also be expressed under normoxic conditions and sEVs derived from those cells can induce a metabolic switch in their surrounding cells [90]. This effect is due to the transfer of glucose transporter GLUT1 and pyruvate kinase PKM2. The transfer of let7a, contained in sEVs derived from hypoxic cancer cells, can increase the oxidative phosphorylation in macrophages [47]. The lncRNA lnc-Ror is enriched in sEVs derived from hypoxic hepatocellular carcinoma cells and its transfer to normoxic cells triggers an increase in HIF-1α levels and a decrease in miR-145 levels in the recipient cells [76]. Consequently, Takahashi et al. also demonstrated that the transfer of lnc-Ror promotes cell survival under hypoxia [76]. Thus, sEVs derived from hypoxic cells can elicit a metabolic switch and hypoxic tolerance in the target cells, which promotes the progression of the cancer cells.

## 3. sEVs from Hypoxic Cells of the Tumor Microenvironment Influence Growth and Migration of Cancer Cells

The TME is modulating the progression of cancer cells and sEVs are known to mediate the communication of cells from the TME and cancer cells [33]. Hypoxic bone-marrow stem cell-released sEVs transfer miR-193a-3p, miR-210-3p and miR-510, which induce EMT in lung cancer cells through STAT3 signaling [91]. sEVs derived from hypoxic mesenchymal stem cells transport miR-21-5p and promote growth and motility of lung cancer cells [85]: miR-21-5p targets PTEN and PDC4 (pyruvate decarboxylase 4), both inhibiting cancer cell growth. Moreover, miR-21-5p down-regulates expression of RECK (reversion-inducing cysteine-rich protein with Kazal motifs), thereby impeding cell motility by inhibiting MMP activity.

Mir-105, which is secreted in sEVs from endothelial cells and which is further upregulated under hypoxic conditions, participates in the destruction of the vascular endothelial barrier. Therefore, it helps the dissemination of cancer cells and subsequent metastasis [92]. Release of mitochondrial DNA (mtDNA) into sEVs derived from fibroblasts can increase oxidative phosphorylation in breast cancer stem-like cells and this transfer of mtDNA also activates self-renewal of breast cancer stem-like cells and expedites their resistance to hormonal therapy [93]. Interestingly, release of mtDNA is induced and increased under hypoxia [94]. These reports all converge on the observation that sEVs released from hypoxic cells in the TME facilitate the progression of cancer.

## 4. sEVs Derived from Hypoxic Cells Promote Resistance to Treatment

Hypoxia has been shown to promote resistance to anticancer drug treatments, and sEVs can play an important role in this process [95,96]. miR-21, by targeting PTEN, promotes resistance to treatment in various cancer types [72,97]. In lung cancer, miR-21, packed in hypoxic sEVs, confers resistance to cisplatin [72]. sEVs derived from hypoxic glioma cells deliver miR-301a, which promotes radiation resistance by downregulating the transcription elongation factor A like 7 (TCEAL7), which is an inhibitor of the β-catenin/T cell factor (TCF) transcription factor, thus leading to the activation of the Wnt/β-catenin signaling pathway [73]. In ovarian cancer, sEVs derived from patient-derived ascites submitted to hypoxia were enriched in STAT3, shown to promote resistance to cisplatin [50].

Resistance to drug treatment can also occur through the efflux of drugs contained in sEVs [98,99,100,101]. This effect could be even aggravated under hypoxic conditions, which trigger an increase of sEV production. However, this remains to be demonstrated and drugs would first have to reach the hypoxic core of the tumor in order to be loaded in sEVs. In addition, multidrug resistance protein 1 (MDR1) gene product P-glycoprotein and multidrug resistance-associated protein-1 (MRP1) can be transferred via sEVs conferring drug resistance to target cells [102,103,104,105]. Hypoxia has been shown to increase P-glycoprotein and MRP1 levels in a HIF-1α -dependent way [106,107,108] and could thus also lead to an elevated level of these proteins in sEVs. Protein arginine methyltransferase 5 (PRMT5), which we have recently found to be enriched in hypoxic melanoma sEVs [22], was shown to be involved in drug resistance against CDK4/6 inhibitors in melanoma [109]. miR-21, in sEVs from cancer-associated fibroblasts, can confer resistance to paclitaxel if transferred to ovarian cancer cells [110] and once again, hypoxia has been demonstrated to induce miR-21 expression [111], potentially also increasing miR-21 levels in hypoxic sEVs. It remains to be seen if those proteins and/or miRNAs upregulated under hypoxia in cell lysates, would also be loaded in hypoxic sEVs, as the proteomes and miRNomes of sEVs often do not completely mirror the proteome and miRNome of the cells from which they are derived [22]. To this date, there are very few studies investigating the role of hypoxic sEVs in promoting or transferring drug resistance, but it is likely to assume that the cargo of hypoxic sEVs is involved in this phenomenon. Toward this end, we have recently identified a truncated form of anaplastic lymphoma kinase (ALK) transported in sEVs of melanoma cells, which conferred drug resistance to BRAF inhibitors by activating the MAPK signaling pathway in target cells [112].

## 5. sEVs Cargo as Potential Biomarkers

sEVs represent a snapshot of the cells of origin and they can be isolated from patients in a non-invasive way, which makes them a versatile reservoir carrying potential biomarkers. The content of hypoxic sEVs could be profiled to indicate stage of disease or possible drug resistance and could thus help in personalizing treatments. In this context, hypoxic sEVs from melanoma cells exhibited a signature consisting of 6 proteins (Aldo-Keto reductase family 7 member A2 (AKR7A2) and DExD-Box Helicase 39B (DDX39B), eukaryotic translation initiation factor 3 subunit C (EIF3C), phenylalanyl-tRNA synthetase subunit alpha (FARSA), protein arginine methyltransferase 5 (PRMT5) and valyl-tNRA synthetase (VARS)), which were significantly associated with a poor prognosis for melanoma patients [22]. However, for routine profiling of sEV content, standardized methods would have to be agreed on allowing for detection of robust biomarkers. A recent study demonstrated detection of sEVs without labeling using an electrochemical sensor, to measure an increase of sEVs secretion under hypoxia from breast cancer cells [113] while the presence of HIF-1α in circulating sEVs was detected via a colorimetric assay [114]. Remarkably, Wang et al. developed gold nanospheres linked to a HIF-1α-binding aptamer in order to allow detection of HIF-1α in the 0.3–200 ng L^−1^ concentration range [114]. Cao et al. identified a sEV-associated gene signature that correlated with intra-tumoral hypoxic status and predicted recurrence in lung adenocarcinoma [115].

miR-210 and miR-1246 have been discussed as biomarkers for glioma and glioblastoma, respectively [66,116]. High levels of miR-210 in serum-derived sEVs were associated with high levels of HIF-1α in glioma patients [116]. In rectal cancer, hypoxia-associated miR-486-5p, miR-181a-5p and miR-30d-5p were enriched in sEVs from sera of 24 patients and identified as circulating indicators of high-risk rectal cancer [117]. The levels of miR-24-3p in sEVs were correlated with poor survival of nasopharyngeal carcinoma patients and therefore, miR-24-3p content in sEVs may serve as a prognostic biomarker for this type of cancer [71]. An increase of miR-885 and a decrease of miR-521 were observed in hypoxic sEVs compared to normoxic sEVs derived from pancreatic cells, and importantly, a similar expression profile was measured in sera from pancreatic cancer patients compared to healthy individuals [118]. LncRNA-p21 was detected in sEVs derived from lung cancer patients and could be used as a biomarker for hypoxic sEVs in non-small cell lung cancer [61]. Apart from their use as biomarkers in cancer, sEV cargos also hold potential in other diseases involving hypoxia: mir-126, loaded in sEVs isolated from sera of ischemic pre-conditioned patients, could indicate a risk of ischemic stroke [119]. These data support the notion that hypoxic sEVs represent a promising reservoir of potential biomarkers for cancer and other diseases. However, it remains to be shown if such biomarkers are reproducible and if expression patterns are robust enough when using different sEV isolation methodologies and protein/miRNA detection techniques and following result validation in independent patient cohorts.

## 6. Conclusions

sEVs exert a plethora of biological functions, ranging from cellular communication to a reshaping of the metabolism and the phenotype of the recipient cell. sEVs derived from hypoxic cancer cells can contribute to an increase of cell proliferation, migration, invasion, EMT and/or angiogenesis. In addition, they are also involved in drug resistance and mediate immunosuppression. Furthermore, hypoxic sEVs assist the cells in their adaptation to hypoxia [120]. Many studies have pointed out the role of miRNAs or proteins carried by hypoxic sEVs in these functions and some recent studies now also describe the involvement of lncRNAs and other ncRNAs. Furthermore, the role of hypoxic sEVs derived from stromal or immune cells from the tumor microenvironment is an area of intense research. Indeed, there is still a lack of evidence whether sEVs produced by hypoxic stromal cells can reshape the behavior of immune cells or vice versa. In this context, the role of hypoxic sEVs derived from immune or stromal cells on the modulation of cancer cells is less well understood. If those sEVs have an important role in tumor progression or regression, it would be of great interest to find strategies/treatments to modulate these responses. In conclusion, sEVs and in particular hypoxic sEVs are important vessels for the transport of bioactive molecules that can exert diverse functions in target cells and tissues. More refined and standardized techniques would aid the identification and comparison of profiling data allowing for better exploration of sEV-derived data sets.

## Figures and Tables

**Table 1:** Proteins enriched in sEVs derived from hypoxic cancer cells.

Protein	Cancer Type	Biological Effects in Cancer	Ref.
C4.4A, α6β4 integrin, MMP14	Pancreatic cancer	Promotes migration	[38]
CD171	Ovarian	Promotes migration	[41]
MMP2, MMP9	Prostate cancer	Enhances invasion	[35]
MMP13	Nasopharyngeal carcinoma	Enhances migration and invasion	[36]
PRMT5	Melanoma	Promotes migration and invasion	[22]
CA9	Renal carcinoma	Enhances migration and angiogenesis	[37]
ADAMTS1, LOX, TSP1, VEGF	Glioblastoma	Promote angiogenesis-related process	[39]
TF	Glioma	Induces angiogenesis	[44]
Wnt4	Colorectal cancer	Induces a pro-metastatic phenotype and angiogenesis	[42,45]
HIF-1α	Nasopharyngeal carcinoma	Promotes EMT-related process	[46]
TGF-β	Prostate cancer	Supports EMT	[35]
CSF-1, CCL2, FTH, FTL and TGF-β	Melanoma, squamous cell carcinoma, lung cancer	Immunosuppressive function	[47]
TGF-β	Lung carcinoma, leukemia, breast cancer	Inhibits NK cells and T cell proliferation	[48,49]
MTA1	Breast cancer	Regulates hypoxia and estrogen signaling	[47]
STAT3	Ovarian cancer	Promotes chemoresistance	[50]

**Table 2:** miRNAs and lncRNAs enriched in small extracellular vesicles derived from hypoxic cancer cells.

miRNA	Target Gene	Cancer Type	Biological Effects in Cancer	Ref.
miR-1290	Not reported	Melanoma	Promotes migration and invasion	[22]
miR-23a	*PHD* and *ZO-1*	Lung cancer	Promotes migration and angiogenesis	[57]
miR-135b	*FIH-1*	Multiple myeloma	Promotes angiogenesis	[56]
miR-155	Not reported	Hepatocellular carcinoma	Increases angiogenesis in endothelial cells	[60]
miR-210	*Ephrin-A3*	Leukemia	Increases angiogenesis	[59]
miR-494	*PTEN*	Non-small lung cancer	Promotes angiogenesis	[58]
miR-1273f	*LHX6*	Hepatocellular carcinoma	Increases proliferation and metastasis	[43]
miR-21	Not reported	Oral squamous carcinoma	Leads to a pro-metastatic phenotype	[24]
miR-10 and miR-21	*Rorα* and *PTEN*	Glioma	Immunosuppressive function towards myeloid derived suppressor cells	[65]
miR-1246	*TERF2IP*	Glioma	Promotes M2 polarization of macrophages	[66]
miR-21-3p, miR-125b-5p and miR-181d-5p	*SOCS4/5/STAT3*	Ovarian cancer	Elicit M2 polarization of macrophages	[67]
miR-301-3p	*PTEN*	Pancreatic cancer	Mediates M2 polarization of macrophages	[68]
miR-940	Not reported	Epithelial ovarian cancer	Promotes M2 polarization of macrophages	[69]
Let7a	*IRS1, IRS2, INSR* and *IGF1R*	Melanoma, squamous cell carcinoma, lung cancer	Elicits M2 polarization of macrophages and increases oxidative phosphorylation	[47]
miR-21	*PTEN*	Oral cancer	Inhibits the expansion and cytotoxicity of γδ T cells	[70]
miR-23a	*CD107*	Lung carcinoma and leukemia	Inhibits NK cells	[48]
miR-24-3p	*FGF11*	Nasopharyngeal carcinoma	Impairs T cell proliferation and differentiation of Th1 and Th17 cells	[71]
miR-21	*PTEN*	Non-small lung cancer	Increases resistance to cisplatin	[72]
miR-301a	*TCEAL7*	Glioma	Activates Wnt/β-catenin pathway and increases resistance to radiation	[73]
Lnc-p21	Not reported	Non-small cell lung cancer	Elicits angiogenesis	[61]
Lnc-UCA1	Not reported	Bladder cancer	Enhances EMT	[74]
LncRNA BRCT1	Not reported	Breast cancer	Promotes M2 polarization of macrophages	[75]
Lnc-Ror	miR-145	Hepatocellular carcinoma	Promotes cell survival under hypoxia	[76]

## Abbreviations

ADAMTS1	A Disintegrin And Metalloproteinase with Thrombospondin Motifs 1
AKR7A2	Aldo-Keto reductase family 7 member A2
ALK	Anaplastic lymphoma kinase
CA9	Carbonic Anhydrase 9
CCL2	C-C motif chemokine 2
circRNA	Circular RNA
CSF−1	Colony-Stimulating Factor 1
CXCL13	C-X-C motif chemokine 13
DDX39B	DExD-Box Helicase 39B
ECM	Extracellular Matrix
EIF3C	Eukaryotic translation Initiation Factor 3 subunit C
EMT	Epthelial to Mesenchymal Transition
eNOS	Endothelial Nitric Oxide Synthase
Erk	Extracellular signal-Regulated Kinase
FARSA	Phenylalanyl-tRNA Synthetase subunit Alpha
FGF11	Fibroblast Growth Factor 11
FIH1	Factor Inhibiting HIF1
FTH	Ferritin Heavy chain
FTL	Ferritin Light chain
HIF	Hypoxia Inducible Factor
HRE	Hypoxia response element
IGF1R	Insulin-like Growth Factor 1 Receptor
IL	Interleukin
ILV	Intraluminal Vesicles
INSR	Insulin Receptor
IRS	Insulin Receptor Substrate
lEVs	Medium/Large Extracellular Vesicles
LHX6	LIM Homeobox 6
lncRNA	Long non-coding RNA
LOX	Protein-lysine 6-oxidase
MCSFR	Macrophage Colony-Stimulating Factor Receptor
MDR1	Multidrug Resistance protein 1
miRNA	Micro-RNA
MMP	Matrix Metalloproteinase
MRP1	Multidrug Resistance-associated Protein−1
MTA1	Metastasis Associated Protein 1
mtDNA	Mitochondrial DNA
MVB	Multivesicular Body
ncRNA	Non-coding RNA
NK	Natural Killer
P-Akt	Phosphorylated Akt
PAR2	Protease Activate Receptor 2
PDC4	Pyruvate Decarboxylase 4
PD-L1	Programmed Death-Ligand 1
PHD	Prolyl Hydroxylase Domain protein
PRMT5	Protein arginine Methyltransferase 5
PTEN	Phosphatase and Tensin homolog
RECK	Reversion-inducing Cysteine-rich protein with Kazal motifs
Rorα	RAR-related Orphan Receptor α
sEVs	Small Extracellular Vesicles
STAT3	Signal Transducer and Activator of Transcription 3
TCEAL7	Transcription Elongation Factor A Like 7
TCF	T Cell Factor
TERF2IP	Telomeric Repeat-Binding Factor 2-Interacting Protein 1
TF	Tissue Factor
TGF-β	Transforming Growth Factor β
TME	Tumour Microenvironment
TNF-α	Tumor Necrosis Factor α
T-reg	Regulatory T cells
TSP1	Thrombospondin−1
VARS	Valyl-tNRA Synthetase
VEGF	Vascular derived Endothelial Growth Factor
VHL	Von Hippel-Lindau
